# Amazon Bioproduct as a Potential Additive for Water-Based Drilling Fluids: Rheological Analysis and Factorial Design Evaluation

**DOI:** 10.1021/acsomega.6c03697

**Published:** 2026-07-14

**Authors:** Thaise do Socorro de Oliveira Araujo, Brenda Fernanda Honorato de Oliveira, Camilo Andres Guerrero-Martin, Emanuele Dutra Valente Duarte

**Affiliations:** † Faculty of Engineering of Exploration and Production of Petroleum, Universidade Federal do Pará − Campus Salinópolis, R. Raimundo Santana da Cruz S/N, Salinópolis 68721-000, Pará, Brazil; ‡ Faculty of Physics, Universidade Federal do Pará − Campus Salinópolis, R. Raimundo Santana da Cruz S/N, Salinópolis, Pará 68721-000, Brazil; § Faculty of Chemistry, Universidade Federal do Pará, R. Augusto Corrêa 01, Belém 66075-110, Pará, Brazil

## Abstract

The need to reuse materials is a global tendency to reduce the amount of waste produced; besides, considering the high operational cost that drilling fluids can offer, ecological fluids have emerged as a low-cost alternative, which also adds value to local biomasses. Therefore, this work studies the compatibility of an Amazon biomass, the Andiroba oil, as an additive for water-based drilling fluids as a rheological modifier. Physical-chemical (density, pH) and rheological parameters (plastic viscosity (PV), yield point (YP), and gel strength­(Gel-10 min). The andiroba-fluid showed values comparable to the standard fluid for density (1.039 g/cm^3^) and pH (9.93); however, the results of the rheological parameters needed to be further addressed. A Full Factorial Design with 3 factors was carried out using andiroba concentration (wt %), salt concentration (NaCl, ppm), and temperature (°C) as variables to optimize the possible conditions for using the chosen biomass, andiroba. Statistical optimization of the results, within the experimental domain studied, showed the optimum point for each parameter to obtain the best PV, YP, and Gel-10 min values that comply with Brazilian legislation, so that the optimum points were: andiroba 1 wt %, NaCl 14,800 ppm, and temperature 78.4 °C. Rheological analysis revealed that the fluids follow a Bingham plastic model at low temperatures (40 °C), while the Herschel–Bulkley model is favored at higher temperatures (80 °C). Therefore, andiroba exhibited an interesting behavior and in accordance with the legislation, presenting itself as an ecologically viable and promising alternative for further studies of fluids based on Amazonian biomasses.

## Graphical abstract

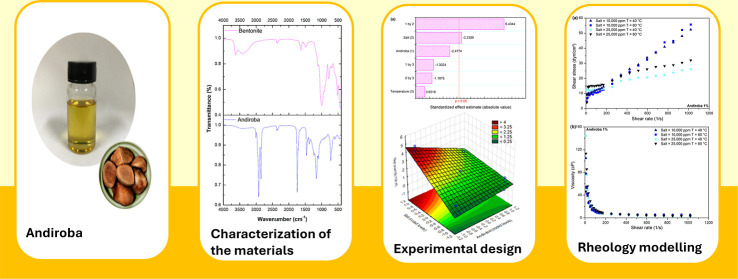

## 1 Introduction

The drilling process of new oil wells is an essential step of oil exploration that must be properly conducted to guarantee the desired production. In this sense, the design of the drilling fluid must include variables that account for the specific characteristics of the formation as well as process functions, such as transport of cuttings, lubrication of equipment, avoiding the influx of fluids from permeable rocks, and formation of a filter cake in the interface well/rock.^1^

The main properties that should be studied in this type of fluid include physical-chemical (density, pH), rheological (plastic viscosity, yield point, and gel point), and filtration (fluid loss control and filter cake) parameters. Among these, the rheological aspects are essential since the behavior of a fluid that mainly follows a Bingham plastic model is desired.^2^ Therefore, understanding the composition and variables that may affect the rheology of drilling fluids is vital.

In general, drilling fluids are a complex mixture that can contain various proportions of solids (dissolved salts, suspended solids, organic material), liquids (water-based or oil-based fluids), and, sometimes, gases. Since drilling fluids can represent 15–18% of the total cost for drilling, they must be easy to use, not too expensive, and environmentally friendly.^3^ Several works have been exploring green alternatives to reduce the environmental impact of drilling fluids, such as the works of: Wang, Liu, and Song,^4^ who investigated a modified product obtained using corn starch as a filtrate control; Le, Duy, and Phung,^5^ by using orange peel powder to modify rheological and filtration properties of a water-based fluid.

New frontiers for oil exploration near the mouth of the Amazon River are enlarging the debate regarding the possible impacts in this region. Considering the great biodiversity and organic variability of biomasses in the Amazon Forest, there is a great opportunity to improve the local bioeconomy by employing Amazon’s biomasses to verticalize the industrial utilization of its resources. Andiroba (*Carapa guianensis*) is a well-known species in the Amazon region and is primarily used in the pharmaceutical and cosmetic industries;^6^ however, since its composition is essentially of fatty acids (oleic, stearic, palmitic, and linoleic),^7^ its oil has potential applications beyond its traditional uses while also contributing to the sustainable utilization of Amazonian natural resources.

In this context, the search for sustainable additives has gained considerable attention, as demonstrated by Ma et al.,^8^ who reported the effectiveness of oleic acid derivatives in reducing friction and improving bentonite dispersion. Given the high oleic acid content naturally present in andiroba oil,^9^ this work proposes an innovative approach by utilizing this Amazonian resource as a rheology modifier and environmentally friendly additive. Unlike some conventional biomass-based additives and other vegetable oils that may exhibit thermal or salinity sensitivity,^10^ the fatty acid-rich chemical composition of andiroba favors interactions with clays and metallic surfaces, potentially enhancing the performance of drilling fluids. Therefore, this study seeks to evaluate the technical feasibility of this Amazonian resource as a sustainable alternative for the development of more efficient and environmentally responsible drilling fluids.

In this sense, this work investigated the innovative employment of Andiroba as a potential additive for rheology modification of water-based drilling fluids. The investigation of physical-chemical and rheological properties of the fluids was performed to visualize the possibilities for utilizing this Amazon’s biomass. A full factorial design with three factors was also included in the investigation to further consider local challenges for oil exploration, such as high concentrations of brine and variations in temperature. The results of this study will open the path for new research using Amazon biomasses, which can aid the community to boost the local bioeconomy, along with reducing possible environmental impacts regarding future oil exploration.

## 2 Experimental Methods

### 2.1 Amazon Biomass

This study utilized Andiroba (*C. guianensis*), which is a biomass that is commonly used and sold at open-air fairs in the northern region of Brazil. The raw material (Figure S1, Supporting Information) used was obtained from a local street market located in the city of Salinópolis/PA (0° 37′ 44″ S, 47° 21′ 21″ O) as an oily material, which was used without any further preparation.

### 2.2 Materials Characterization

The biomass (Andiroba), bentonite, and the muds developed were analyzed using Fourier transform infrared spectroscopy (FTIR) in order to identify the main composition of the materials used. The analyses were carried out on a spectrometer (Bruker, Vertex 70v) in the attenuated total reflectance (ATR) module under the following conditions: scanning in the 400 to 4000 cm^–1^ region, 45 scans, and 4 cm^–1^ resolution.

### 2.3 Drilling Fluid Preparation and Analysis

The fluid samples were prepared using a standard 6 wt % bentonite base, equivalent to 15 g (EVEN Analytical Balance, FA-2204B), to a total of 250 g of solution using distilled water as the solvent. The fluids with the biomass additive were prepared by adding 1% Amazonian biomass to the standard fluid, equivalent to 2.5 g per 250 g. The mixtures were homogenized in a magnetic stirrer (Lucadena, Luca −01/09) for 15 min.

The fluids were analyzed using density, pH, and rheological parameters. Density was obtained using the pycnometer method, while pH was checked using a previously calibrated pH meter (HANNA, FT-P21). The rheological analyses were carried out on a viscometer (Grace, M3600) according to API RP 13B-1 (American Petroleum Institute Recommended practice),^11^ in duplicate, under the following conditions: (i) determination of plastic viscosity (PV) and minimum yield stress (YP)—rotation from 0 to 600 rpm at 25 °C and ambient pressure—and (ii) determination of the gel point—sample prestabilization at 600 rpm during 5 min, followed of a nonagitation period of 10 s or 10 min and then by rotation at 3 rpm, at 25 °C and ambient pressure. The PV and YP values were determined using eqs 1 and 2, respectively.

1 $$PV=\theta_{600}-\theta_{300}$$

2 $$YP=\theta_{300}-PV$$

where PV is the plastic viscosity (cP), θ_600_ is the dial reading at 600 rpm, θ_300_ is the dial reading at 300 rpm, and YP is the minimum yield stress (lb/100 ft^2^).

The fluids were evaluated regarding their rheological behavior using the Power law (eq 3), Herschel–Bulkley (eq 4), and Bingham Plastic (eq 5) models. The models were statistically evaluated using the coefficient of determination (*R* ^2^), the adjusted coefficient of determination (*R* ^2^_adjust_), the Root mean squared error (RMSE), and Akaike Information Criterion (AIC), as shown in eqs 6, 7, 8, and 9, respectively.

3 $$\tau =K{\mathrm{\gamma ̇}}^{n}$$

4 $$\tau =\tau_{y}+K{\mathrm{\gamma ̇}}^{n}$$

5 $$\tau =\tau_{y}+\mu_{P}\mathrm{\gamma ̇}$$

6 $$R^{2}=\frac{SSM}{SST}=1-\frac{RSS}{SST}$$

7 $$R_{adjust}^{2}=1-\left(\frac{SSM}{SST}\right)\left(\frac{m-1}{m-p-1}\right)=1-\left(1-R^{2}\right)\left(\frac{m-1}{m-p-1}\right)$$

8 $$RMSE=\sqrt{\frac{1}{m}\sum_{i=1}^{m}{\left(y_{i}-\hat{y_{i}\;}\right)}^{2}}$$

9 $$AIC=\{\begin{array}{l} m\times \ln\left(\frac{RSS}{m}\right)+2p,,when\frac{m}{p}\geq 40\\ m\times \ln\left(\frac{RSS}{m}\right)+2p+\frac{2p\left(p+1\right)}{m-p-1},,when\frac{m}{p}<40 \end{array}$$

where τ is the shear stress (lb/100 ft^2^); γ̇ is the shear rate (s^–1^); μ_P_ is the plastic viscosity (cP); *K* is the consistency coefficient (*s* ^*n*^ × lb/100 ft^2^); *n* is the flow behavior index (dimensionless); τ_*y*_ is the yield stress (lb/100 ft^2^); SSM is the sum of squares of the model; RSS is the residual sum of squares; SST is the total sum of squares; *m* is the number of observations; *p* is the number of model parameters; *y*_*i*_ is the *i*th observed value; and $\hat{y_{i}\;}$ is the *i*th value calculated by the model.

### 2.4 Experimental Design Analysis

Recently, there have been studies in Brazil considering the implementation of oil exploration near the Foz do Amazonas Basin, which is a saltwater region where the Amazon River encounters the Atlantic Ocean.^12^ Considering the possibility of utilizing ocean water for formulating a drilling fluid, this study evaluated the impact of salt concentration and temperature variation on drilling fluids produced with bentonite by employing Andiroba oil, an Amazon subproduct, as an additive. This was performed by using a 2^3^ factorial design with two central points, giving a total of 10 analyses. The factors and levels that were studied are shown in Table 1, which show the coded and actual levels of each parameter used in the analyses.

**Table 1:** Variables and Factors Utilized in This Experimental Design—Real and Coded Levels

	factors		
variables	–1	0	1
Andiroba (wt %)	1.0	1.5	2.0
[NaCl] (ppm)	10,000	17,500	25,000
temperature (°C)	40	60	80

The concentration of andiroba was determined considering that, in the first round of analyses, the employed value of 1 wt % returned good results, so it was considered that an increase in concentration would enhance the performance of the drilling fluid.

The temperature of the well is an important parameter that may affect the behavior of the mud; thus, it was included as a variable in this study. Cavalcante, de Argollo, and Carvalho^13^ evaluated downhole temperatures in the sedimentary basins of the Bahia region (northeast of Brazil), identifying temperatures in the range between 40 and 160 °C. Taking this into account, it was decided to use a minimum temperature value of 40 °C. The maximum temperature was defined considering the limitations of the equipment used in the viscosity analysis, which cannot exceed 100 °C. It was therefore decided to carry out the evaluation between 40 and 80 °C.

Salmachi, Talemi, and Tooski^14^ described the influence of salt concentration in drilling fluid and reported that a salt concentration above the seawater level (35,000 ppm) has an impact on the bentonite present in the fluid, rendering it inert. Furthermore, the authors state that the use of sodium chloride concentration in water from zero (distilled water) to 10,000 ppm markedly reduces the apparent viscosity of the mud, which is a common observation for many viscosity agents. The tests carried out by the authors showed that the apparent viscosity of the mud remains almost unchanged for further increases in the concentration of sodium chloride above 25,000 ppm. Therefore, based on the literature, the region of analysis used for this factorial design was between 10,000 and 25,000 ppm, a region in which it was hoped to observe a greater impact on the results for application in this work.

For the response variables analyzed, the parameters initially evaluated were plastic viscosity (PV), yield strength (YP), initial gel strength (Gel-10s), final gel strength (Gel-10 min), pH, and density. These parameters were chosen, because they are routinely measured in field operations. The analysis of the results identified that all of the values for Gel-10s, pH, and density calculated for the samples were in accordance with the Brazilian legislation; thus, only the parameters that would have a significant impact on the investigation were maintained in the statistical evaluation of the factorial design. The tests were carried out according to plan 2^3^, which is shown in Table 2, and were performed randomly.

**Table 2:** Matrix of the 2^3^ Full Factorial Experimental Design Used for This Study

	coded levels			real levels		
analysis	Andiroba	salt	temperature	sndiroba concent. (wt %)	salt concent. (ppm)	temperature (°C)
1	1	–1	–1	2.0	10,000	40
2	+1	+1	+1	2.0	25,000	80
3	–1	–1	+1	1.0	10,000	80
4	–1	+1	–1	1.0	25,000	40
5	–1	–1	–1	1.0	10,000	40
6	+1	+1	–1	2.0	25,000	40
7	+1	–1	+1	2.0	10,000	80
8	–1	+1	+1	1.0	25,000	80
9	0	0	0	1.5	17,500	60
10	0	0	0	1.5	17,500	60

## 3 Results and Discussion

### 3.1 Characterization of the Biomass and the Produced Fluid

Figure 1 shows the results of the FTIR analyses of the raw materials used. Bentonite is a silicate-based clay from the smectite group that typically presents Al, Mg, and Si in its composition. The curve on Figure 2 for bentonite shows bands related to the Al–Al–OH group, at 3626.04 cm^–1^ due to stretching and at 912 cm^–1^ due to bending; presence of structural water, identified by O–H stretching (3384.94 cm^–1^) and H–O–H bending (1637.50 cm^–1^); Si–O stretching, at 1105.17 and 997.16 cm^–1^; Al–OH–Mg bending band at 879.51 cm^–1^; 777.28 cm^–1^ and 692.41 cm^–1^ bands that are probably related to the presence of quartz and dolomite in the sample, respectively; and finally Al–O–Si stretching at 511.12 cm–1 ^15,16^

**Figure 1:**
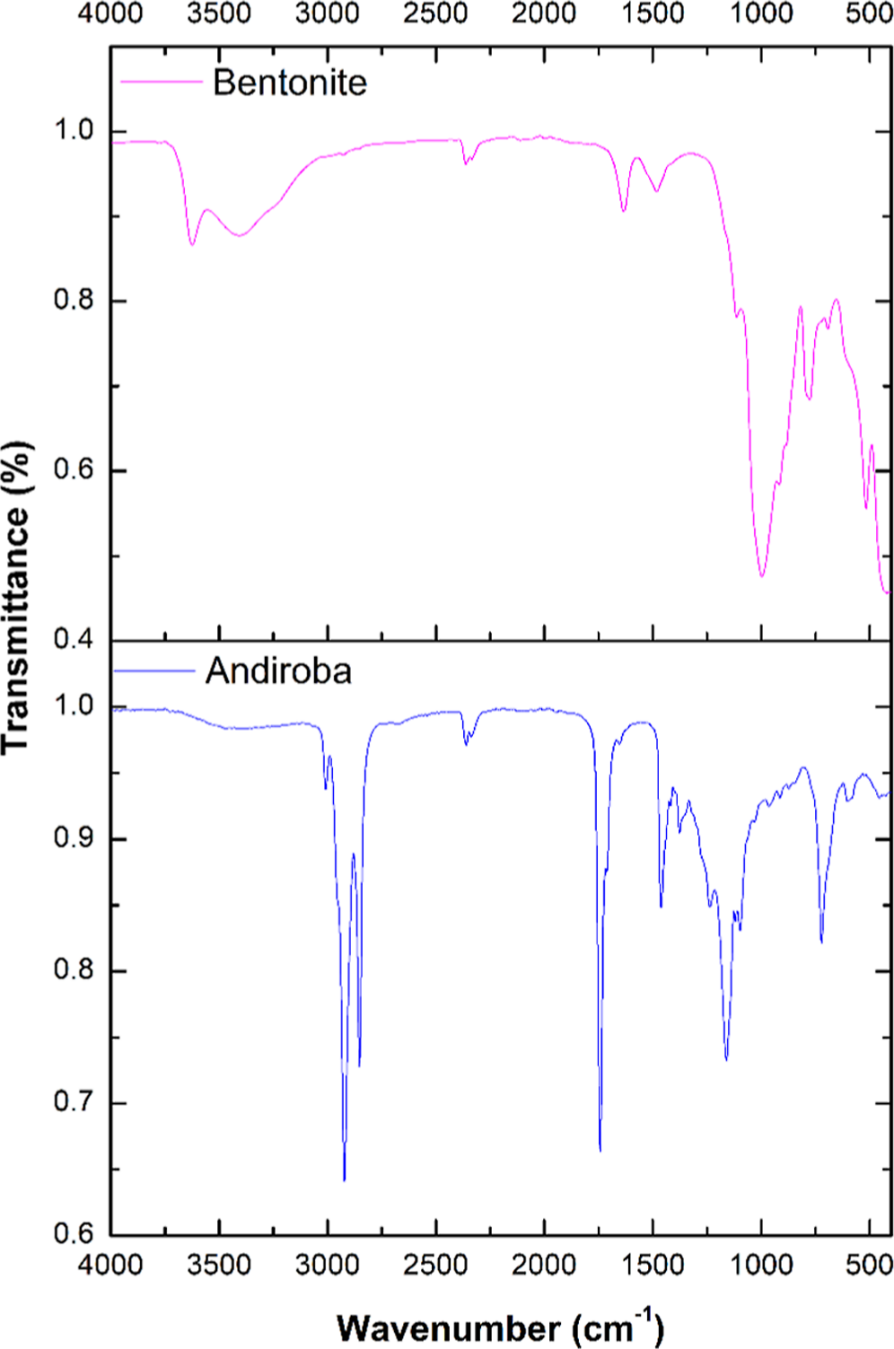
FTIR analyses of the used raw materials.

**Figure 2:**
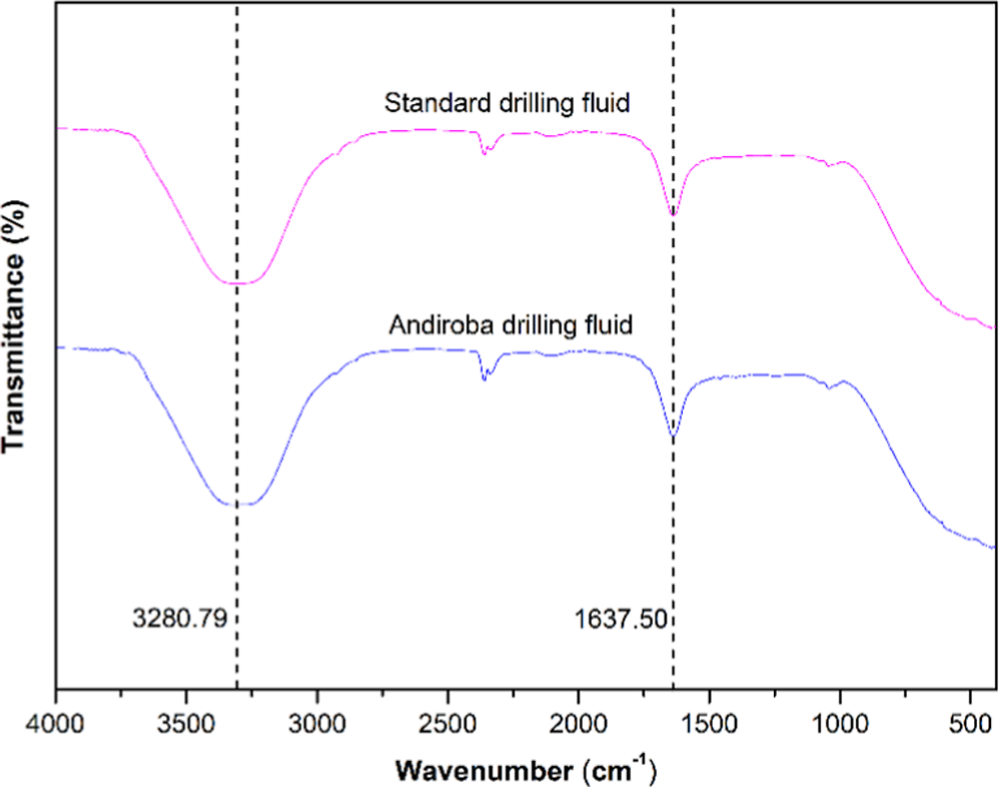
FTIR analysis of the produced drilling fluids.

As for the biomass (andiroba), its composition is predominantly organic matter, which can be identified by the presence of bands related to C–H asymmetric and symmetric stretching (2923.98 and 2854.54 cm^–1^), C=O stretching (1743.58 cm^–1^), C–H scissoring bending (1463.92 cm^–1^), C–O stretching (1163.03 cm^–1^), and C=C bending (723.28 cm^–1^). The curve pattern shown in Figure 1 is consistent with spectra available in the literature for andiroba.^17^

After the drilling fluids were produced, the spectra obtained (Figure 2) resulted in the same curve regardless of whether the additive was added or not. This is because the concentration of additives used is low (only 1 wt %), which is not enough to have an impact on the vibrational frequency of the groups present in the fluid. In this sense, the relevant bands for all the muds developed are O–H stretching (3280.79 cm^–1^) and H–O–H bending (1637.50 cm^–1^), which is related to the large amount of solvent used (distilled water).

Table 3 presents the parameters obtained for the andiroba-based drilling fluid along with the values for the standard fluid (bentonite only). It can be observed that although the andiroba-prepared fluid is largely in compliance with national industry standards (N-2604)^18^ and the International Literature Benchmark (API-based), the identification of certain parameters requiring optimization served as the foundation for the subsequent stage of this study. This stage focuses on an experimental design to refine the formulation and maximize the potential of the andiroba oil as an additive.

**Table 3:** Properties of the Drilling Fluids with and without the Addition of the Amazon Biomass

drilling fluid	gel-10s (lb/100 ft^2^)	gel-10 min (lb/100 ft^2^)	PV (cP)	YP (lb/100 ft^2^)	YP/PV	density (g/cm^3^)	pH
standard	9.78	8.02	6.75	13.89	2.06	1.054	10.13
andiroba	6.06	7.43	4.21	9.78	2.32	1.039	9.93
brazilian legislation	>7	10 to 15	>4	<15 × PV	<15	1 to 2	7 to 10
literature benchmark (API-based)^19–22^	2–15	5 to 28	>4	5 to 42	<3	1 to 1.4	8 to 11

The search for more sustainable additives with lower environmental impact for water-based drilling fluids has driven research into biopolymers and natural materials. Although the present study focuses on the evaluation of andiroba oil against a standard bentonite system, recent literature validates the potential of Amazonian biopolymers and biomasses as alternatives or complements to commercial additives. da Costa et al.^23^ demonstrated that *Opuntia cochenillifera* mucilage exhibits rheological synergy with xanthan gum, increasing apparent viscosity by up to 70% and maintaining stability in high salinity. Complementarily, Azevedo et al.^24^ showed that the integration of açaí ashes into commercial formulations improves plastic viscosity by 30%. These studies establish a precedent for using regional resources as technically effective additives, positioning the initial characterization of andiroba as a fundamental step for future optimizations and direct comparisons with polymers such as CMC, PAC, and xanthan gum.

### 3.2 Experimental Planning

A full factorial design with 3 factors, 2 levels, and 2 central points was designed to evaluate the efficiency and impacts of predefined parameters (temperature, andiroba concentration, and salt concentration) on the drilling fluid. The preparation of the fluids was based on the statistical planning described in Table 2 (Section 2.4). Table 4 shows the factorial design matrix and the results found for plastic viscosity (PV), yield point (YP), Gel-10s, Gel-10 min, pH, and density, which were obtained for the prepared fluids.

**Table 4:** Results of the Factorial Design

sample	andiroba	salt	temperature	gel-10s (lb/100 ft^2^)	gel-10 min (lb/100 ft^2^)	PV (cP)	YP (lb/100 ft^2^)	density (g/cm^3^)	pH
1	–1	–1	1	9.98	12.98	5.71	0.00	1.058	7.80
2	+1	–1	–1	6.98	5.98	1.40	1.50	1.019	7.84
3	–1	+1	–1	12.98	6.98	1.01	3.20	1.060	8.29
4	+1	+1	+1	5.98	7.98	2.38	1.63	1.018	8.13
5	–1	–1	–1	10.98	8.98	4.23	0.95	1.055	8.01
6	+1	+1	–1	6.98	7.98	2.90	1.63	1.019	7.80
7	+1	–1	+1	2.98	2.98	1.40	0.00	1.054	7.69
8	–1	+1	+1	9.98	10.98	1.10	4.87	1.022	8.16
9	0	0	0	7.98	7.98	1.60	2.09	1.063	7.65
10	0	0	0	7.98	7.98	2.05	1.37	1.018	7.70

The results obtained from the factorial design aid in understanding the impact that the tested variables have on the drilling fluids. From the data obtained, the parameters that had a significant change in the fluid’s behavior were identified, namely, plastic viscosity, yield point, and Gel-10 min. According to Andrade,^25^ plastic viscosity, yield point, and gel point are rheological parameters of the drilling fluid that will directly influence the calculation of load losses in the pipe and the transportation speed of the residual drilling gravels. Thereafter, these parameters play a crucial role in the characterization and performance of the fluid in question, so they were chosen as response variables of interest for optimization, as they are necessary for determining the properties and behavior of the fluid in various applications. Statistical optimization was used to find the optimum settings for each of these parameters in order to improve the performance of the solutions in question. The other parameters (Gel-10s, density, and pH) are mainly included within the interval defined as well-conform according to the Brazilian legislation^18^ and API values;^19–22^ thus, these parameters were not included in the next steps of the statistical analysis.

#### 3.2.1 Estimated Effects

Considering a confidence level of 95% (*p* <0.05), Table 5 shows the influential variables for each of the responses (PV, YP, and Gel-10 min). Initially, it can be seen that the mean/intercept is significant for all dependent variables—this is related to the fact that, even when andiroba and salt concentrations are null, the standard drilling fluid has its own rheological properties, as expected and detailed previously in Table 3.

**Table 5:** Estimated Effects of the Results for This Study^a^

factor	PV			YP			gel-10 min		
	effect	standard error	*p*	effect	standard error	*p*	effect	standard error	*p*
mean/Interc	2.3794	0.1791	0.0009	1.7235	0.1175	0.0007	8.0751	0.1959	0.0000(3)
andiroba (1)	–0.9920	0.4004	0.0895	–1.0630	0.2628	0.0057	–3.7500	0.4380	0.0033
NaCl (2)	–1.3370	0.4004	0.0444	2.2175	0.2628	0.0035	0.7498	0.4380	0.1854
temperature (3)	0.26100	0.4004	0.5410	–0.1960	0.2628	0.5098	1.2500	0.4380	0.0649
1 by 2	2.5765	0.4004	0.0076	–1.3375	0.2628	0.0147	2.7500	0.4380	0.0082
1 by 3	–0.5215	0.4004	0.2838	–0.5550	0.2628	0.1251	–2.7503	0.4380	0.0082
2 by 3	–0.4755	0.4004	0.3205	1.0305	0.2628	0.0295	0.7500	0.4380	0.1853

a The 5th significant number is shown in parentheses whenever it is necessary.

Among the described parameters, the effect of increasing the amount of NaCl is detrimental to PV, making it an influential and negative variable, as expected; on the other hand, the interaction between variables 1 and 2 (andiroba and NaCl) has a positive impact on PV, making it an influential, positive, and desirable effect. For the relationship in the YP parameter, the variable that has the greatest impact is NaCl concentration, which has an influential, positive, and desirable effect; besides, the correlation between variables 1 and 2 (andiroba and NaCl) together and the andiroba alone defines influential, negative, and undesirable effects; finally, the correlation between salt and temperature (2 by 3) has an influential and positive effect on the produced fluid, thus, it is obvious that these variables interact in a nonlinear behavior with respect to the YP response. For Gel-10 min, andiroba and the correlation between variables 1 and 3 (andiroba versus temperature) have a negative and undesirable effect, while the correlation between variables 1 and 2 (andiroba and NaCl) represents a positive and desirable effect.

#### 3.2.2 Pareto Diagram

The Pareto diagram identifies the order of influence of the variables on the results by standardization of the effect, making it possible to determine the most relevant interactions between the factors and which of them are most significant for the response.^26^ The *p*-value = 0.05 on the Pareto chart shows the limit of significance, so that below the red line of “*p* = 0.05”, the variable can be considered insignificant or without influence on the experiment at a confidence level of 95%.^27^ The Pareto diagram for this study is exhibited in Figure S2 (Supporting Information).

Figure S2a shows that it is possible to change the value of PV by varying the interaction between factors 1 and 2 (andiroba and NaCl) and the salt concentration. Figure S2b exhibits that it is possible to modify the value of YP by varying (in descending order) the salt concentration, the interaction of factor 1 by 2 (andiroba and NaCl), the andiroba concentration, and finally the correlation between factors 2 and 3 (NaCl and temperature). Figure S2c for Gel-10 min shows that it is possible to alter this response by varying the andiroba concentration, the interaction of factors 1 by 3 (andiroba and temperature), and the interaction between variables 1 and 2 (andiroba and NaCl). Therefore, the models obtained to represent the response variables based on the studied independent variables can be mainly reduced to contain only significant interactions, as identified in both the estimated effects and Pareto analyses.

#### 3.2.3 Analysis of Variance (ANOVA)

Table 6 shows the analysis of variance (ANOVA), which is a statistical technique that separates the sources of variability in the data between the variance related to the model and the variance due to experimental error (residual error) in the analysis, to check whether the error related to the model generated is significant compared to the residual error.^28,29^

**Table 6:** Analysis of Variance (ANOVA) for the Studied Factorial Design with the Values for PV, YP, and Gel-10 min

		PV				YP				gel-10 min			
factor	df^a^	SS^b^	MS^c^	*F* ^d^	*p* ^e^	SS^b^	MS^c^	*F* ^d^	*p* ^e^	SS^b^	MS^c^	*F* ^d^	*p* ^e^
andiroba (1)	1	1.9681	1.9681	6.1373	0.0895	2.2599	2.2599	16.3666	0.0272	28.1250	28.1250	73.3186	0.0033
NaCl (2)	1	3.5751	3.5751	11.1485	0.0444	9.8346	9.8346	71.2228	0.0035	1.1243	1.1243	2.9308	0.1854
temperature (3)	1	0.1362	0.1362	0.4249	0.5610	0.0768	0.0768	0.5564	0.5098	3.1250	3.1250	8.1465	0.0649
1 by 2	1	13.2767	13.2767	41.4014	0.0076	3.5778	3.5778	25.9107	0.0147	15.1250	15.1250	39.4291	0.0082
1 by 3	1	0.5439	0.5439	1.6962	0.2838	0.6161	0.6161	4.4615	0.1251	15.1278	15.1278	39.4363	0.0082
2 by 3	1	0.4522	0.4522	1.4101	0.3205	2.1239	2.1239	15.3811	0.0295	1.1250	1.1250	2.9327	0.1853
residual error	3	0.9621	0.3207			0.4143	0.1381			1.1508	0.3836		
SS^b^ total	9	20.9144				18.9034				64.9028			

a df: degrees of freedom.

b SS: sum of squares.

c MS: mean of squares.

d *F*: Fisher’s value.

e *p*: probability of significance.

The ANOVA (Table 6) shows the variables with the lowest probability of remaining in the acceptance range of the null hypothesis, at the 95% confidence level (p <0.05). The adequacy of the model based on the significant factors is verified by its Fisher’s value (ratio between the mean squared error of the model and the mean squared error of the residuals): if *F*_model/error_ is greater than *F*_tabulated_ (for the same degrees of freedom at the predefined α level), then the developed model is acceptable.^30^ Table S1 shows that the three complete models well-represent the phenomenon since *F*_model/error_ > *F*_tabulated_; however, it is clear from the analyses of estimated effects (Section 3.2.1) and Pareto (Section 3.2.2) that reduced models may be sufficient for calculating the response variables.

Therefore, to build the reduced models, the backward elimination methodology was applied and identified the factors that did not highly impact the calculated results for each response, so these nonsignificant factors were discarded. In this way, it was possible to either keep the *R* ^2^ and *R* ^2^_adjust_ values of the reduced models close to unity and to acquire representative equations with *F*_model/error_ > *F*_tabulated_. Besides, it was also considered the adequacy of the residual plots during the development of the reduced models, therefore, some interactions were kept guaranteeing the assumptions of independence, normality, and homoscedasticity of residuals, as detailed in the next section.

Thus, the reduced models are represented in eqs 10, 11, and 12 for PV, YP, and Gel-10 min (respectively) using the following acronyms for each parameter: *X*1 (andiroba concentration), *X*2 (Salt concentration), and *T* (Temperature). The calculated Fisher’s values for each reduced model are available on Table S2 and highlight that these models are representative of the responses. Moreover, the determination coefficients calculated for the reduced models using coded variables showed great adequacies, with values of: (i) for PV, *R* ^2^ = 0.9259, *R* ^2^_adjust_ = 0.8666, and RMSE = 0.5569; (ii) for YP, *R* ^2^ = 0.9414, *R* ^2^_adjust_ = 0.8946, and RMSE = 0.4708; and (iii) for Gel-10 min, *R* ^2^ = 0.8995, *R* ^2^_adjust_ = 0.8492, and RMSE = 1.0438.

10 PV=2.3794−0.4960×X1−0.6685×X2+1.2883×X1×X2−0.2608×X1×T

11 YP=1.7235−0.5315×X1+1.1088×X2−0.6688×X1×X2+0.5153×X2×T

12 $${Gel}_{10\min}=8.0751-1.8750\times X1+1.3750\times X1\times X2-1.3751\times X1\times T$$

The developed models highlight that as expected, the rheological parameters of the drilling fluids are dependent on the tested input variables and can be estimated by the combination of this multivariate system. Therefore, a fluid that encompasses all the desired characteristics as predefined by the legislation can be designed by the proper combination of the representative models in a more straightforward manner, which takes less time and effort when compared to the alternative of performing several analyses to determine the values of *X*1, *X*2, and *T*. Section 3.2.6 will discuss greater on the calculation of the combination of these values.

#### 3.2.4 Error Analysis for Model Prediction

This study presented three reduced models to predict the response variables of Gel-10 min, PV, and YP. In order to evaluate the reliability of the proposed models, three analyses were conducted: (i) residual analysis; (ii) statistics of the model; and (iii) uncertainty of prediction.

According to Montgomery,^26^ the analysis of variance hypothesizes that the model errors (residuals) are normally and independently distributed; besides, it is also expected that they possess homoscedasticity. To verify these assumptions, the residuals were calculated according to eq 13 for each model and three plots were constructed: (a) residuals vs observations order, used to identify the independence assumption, which is made by observing if residuals are randomly distributed; (b) residuals vs predicted values, employed to verify if residuals possess constant variance (homoscedasticity) and no observed pattern; and (c) *Q*–*Q* plot, utilized to confirm that residuals follow a normal distribution. Figures S3, S4, and S5 provide these plots for PV, YP, and Gel-10 min, respectively. These plots indicate that the residuals of the reduced models are in accordance with the assumptions of the ANOVA, suggesting that there was no bias during model development.

13 $$e=y_{o,i}-y_{p,i}$$

where *e* is the error; *y*_*o*,*i*_ is the *i*th observed value; and *y*_*p*,*i*_ is the *i*th predicted value.

Regarding the statistical values of the models, as indicated in the previous section, the models presented values of 0.8995 < *R* ^2^ < 0.9414, 0.8492 < *R* ^2^_adjust_ < 0.8946, and 0.4708 < RMSE <1.0438. These results indicate satisfactory agreement between experimental and predicted responses within the investigated experimental domain, supporting the adequacy of the fitted model. Furthermore, the relatively low RMSE values observed for all of the models indicate satisfactory experimental consistency and reproducibility within the investigated experimental domain.

To evaluate parameter uncertainty and model reliability, Table S3 presents the confidence interval of the coefficients for each model, the probability of significance for each term, and the standard error of the coefficients. As can be seen, the majority of the coefficients for the proposed reduced models were statistically significant at a 95% confidence level (since *p* <0.05), indicating that the investigated factors and interactions have a significant influence on the response. Besides, the standard errors associated with the estimated coefficients were low (0.1489–0.3687) for all models, indicating good precision in the estimated parameters.

With all this considered, it is important to notice that, although the factorial design was performed with only ten experiments, the statistical analysis of the variance demonstrated adequate models’ performance. Considering that the investigated factor intervals were selected based on preliminary screening experiments and practical operational limits associated with drilling fluid formulation, as explained in detail in section 2.4 of methodology, the developed model should be interpreted as valid only within the studied experimental domain, and extrapolation beyond these ranges may increase uncertainty. Additional validation experiments could further strengthen the predictive capability of the model and will be considered in future studies.

#### 3.2.5 Response Surface

Response surface graphs were used to investigate the trend of optimization of the analysis of the results of each parameter in this study. The surface graph makes it possible to visualize the region in which there are greater or lesser results in the desired response based on the change in two variables. As three variables were used in this study, the value of the third variable was fixed at the center point to generate the plots.

Plastic viscosity (PV) can be defined as a measure of the fluid’s internal resistance to flow, which results from the interaction of solids that are present in suspension. For fluids based on water and bentonite clays, Petrobras^18^ specifies in standard N-2604/1998 that the plastic viscosity value must be at least 4.0 cP, which is in accordance with international values from API. In Figure 3a, the dark-red region of the graph represents the desired area, which is observed when both andiroba and salt concentrations are near their respective lower factorial values (1.0 wt % for andiroba and 10,000 ppm for salt). Additional response surfaces for PV are not shown since temperature is not an influential factor for this response variable.

**Figure 3:**
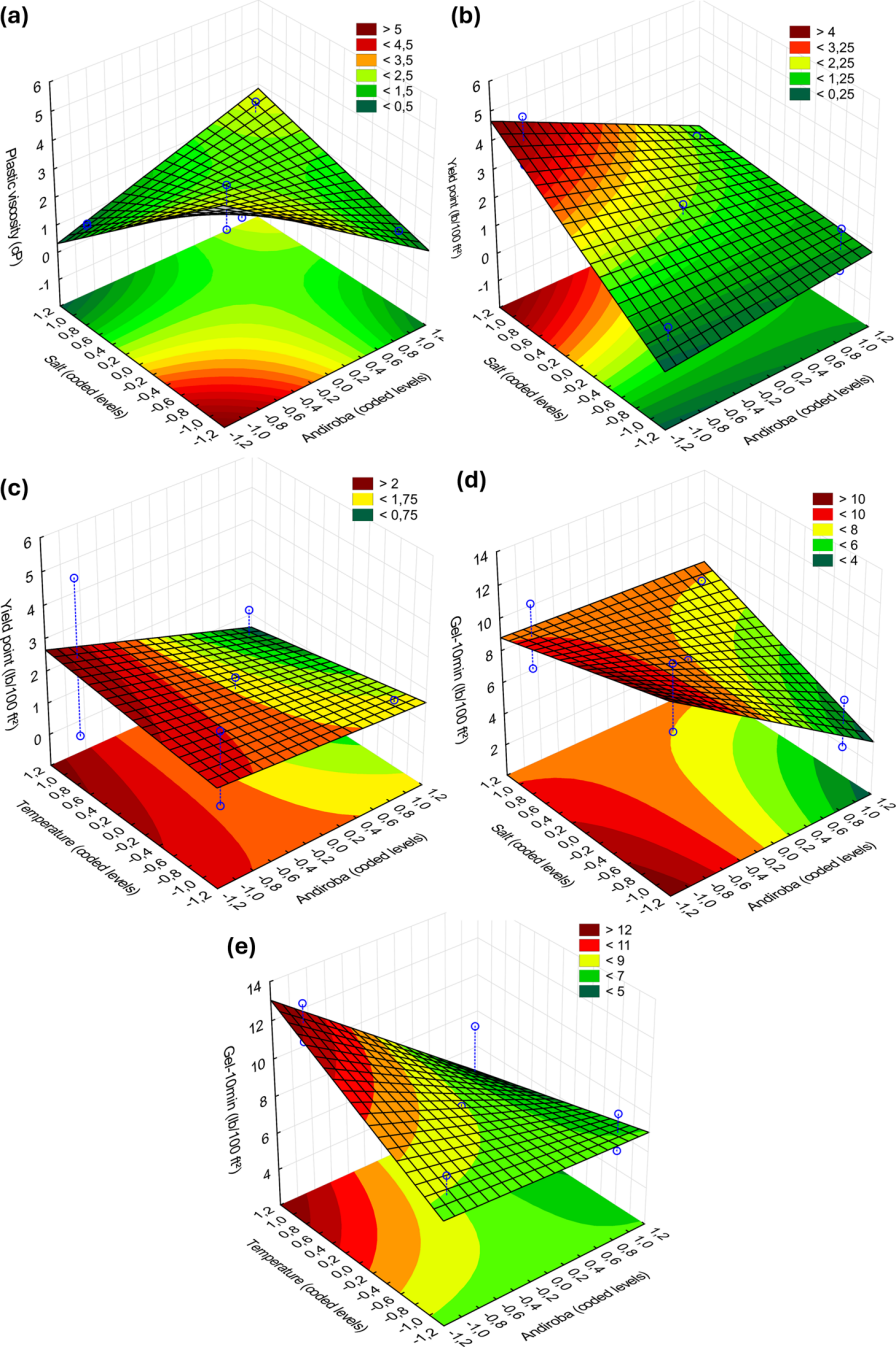
Surface plots for the interaction of: (a) salt and andiroba into PV; (b) salt and andiroba into YP; (c) temperature and andiroba into YP; (d) salt and andiroba into Gel-10 min; and (e) temperature and andiroba into Gel-10 min. The third variable was set at the center point.

The yield point (YP) is particularly important for drilling fluids, since a higher value of this parameter is related to the mud’s better ability to carry gravel to the surface. However, excessively high values require greater pump power and operating costs.^31^ In N-2604/1998 of the Brazilian legislation, the maximum value for YP is 15 × PV. In Figure 3b, within the region studied, all the values are in accordance with the legislation (YP < 15 × PV), but considering that the YP value of the standard sample is 13.89 lb/100 ft^2^, it is suggested that the YP to be maintained at least above 2.00 lb/100 ft^2^ (orange region), which ranges from 1.0 to 1.3 wt % for andiroba (in the figure, coded levels −1.0 and −0.4, respectively) and from 17,000 to 25,000 ppm for NaCl (in the figure, coded levels −0.4 and −1.0, respectively). Figure 3 c shows that temperature has no direct influence on YP, since values of YP >2.00 lb/100 ft^2^ can be achieved whenever andiroba concentration is between 1.0 and 1.2 wt %, while the temperature range is within the studied interval (40 to 80 °C), as represented by the red regions. As for international standards, since 5 ≤ YP ≤ 42 lb/100 ft^2^, further analyses are required in another domain interval to attend the API benchmark values.

The purpose of the gel tests is to assess the fluid’s ability to hold the debris at different times (after 10 s and 10 min) when drilling is interrupted. In this way, it is possible to check whether the fluid’s rheological response is fast or slow when drilling begins after a possible pause.^32^ According to the N-2604/1998 of Petrobras, the range for Gel-10 min is from 10 to 15 lb/100 ft^2^, which is in accordance with API values of 5 to 28 lb/100 ft^2^. When the gel values are too high, they become undesirable, as returning to drilling after a stoppage would require a great effort from the drill bit; on the other hand, when gel values are much lower than those stipulated, they can end up clogging the well due to the debris not being supported by the gel.^32^ Figures 3d and e show the response surface regions for Gel-10 min, where it is recommended to keep the fluid in the region marked by the red color in both graphs to maintain the fluid between the regulatory values. In this case, it is clear that the concentration of andiroba should be kept at a minimum (∼1 wt %) associated with a temperature above 60 °C (Figure 3e), regardless of the value of salt concentration (Figure 3d).

#### 3.2.6 Desirability

This analysis was carried out to ensure that all 3 parameters of interest (PV, YP, and Gel-10 min) were simultaneously in accordance with the values in the Brazilian legislation. The approach that uses the desirability function represents a technique that allows you to simultaneously determine the ideal configurations of the input variables capable of influencing the desired performance levels for one or more responses. The desirability procedure comprises two fundamental steps: (1) identifying the values of the independent variables that simultaneously produce the most desirable predicted responses in the dependent variables and (2) optimizing the overall desirability by taking into account the factors that can be controlled.^33^ Thereafter, as displayed in Figure 4, the values of andiroba, salt, and temperature required to achieve the PV, YP, and Gel-10 min levels in accordance with the legislation were: 1% of andiroba (on the image, coded level −1), 14,800 ppm of salt (coded level −0.36), and 78.4 °C (coded level 0.92). The values calculated are within the scope of this study.

**Figure 4:**
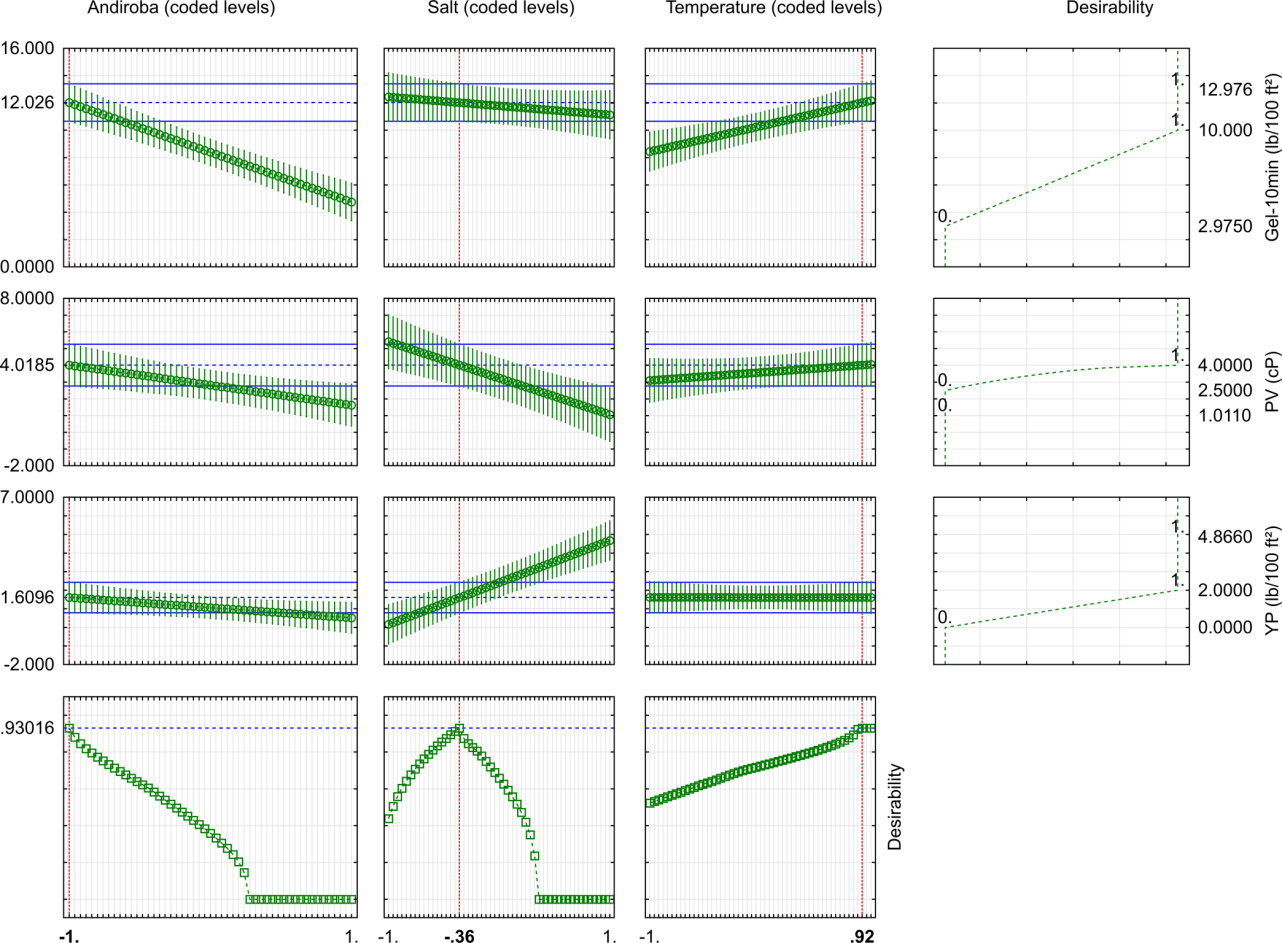
General desirability for optimizing PV, YP, and Gel-10 min simultaneously.

### 3.3 Rheology of the Drilling Fluids with Andiroba

The experiments performed during the factorial design generated shear stress and viscosity profiles that were subdivided into andiroba concentrations of 1 wt % (Figure 5a and b) and 2 wt % (Figure 5c,d). The results of rheology modeling are displayed in Tables S4 and S5 (Supporting Information).

**Figure 5:**
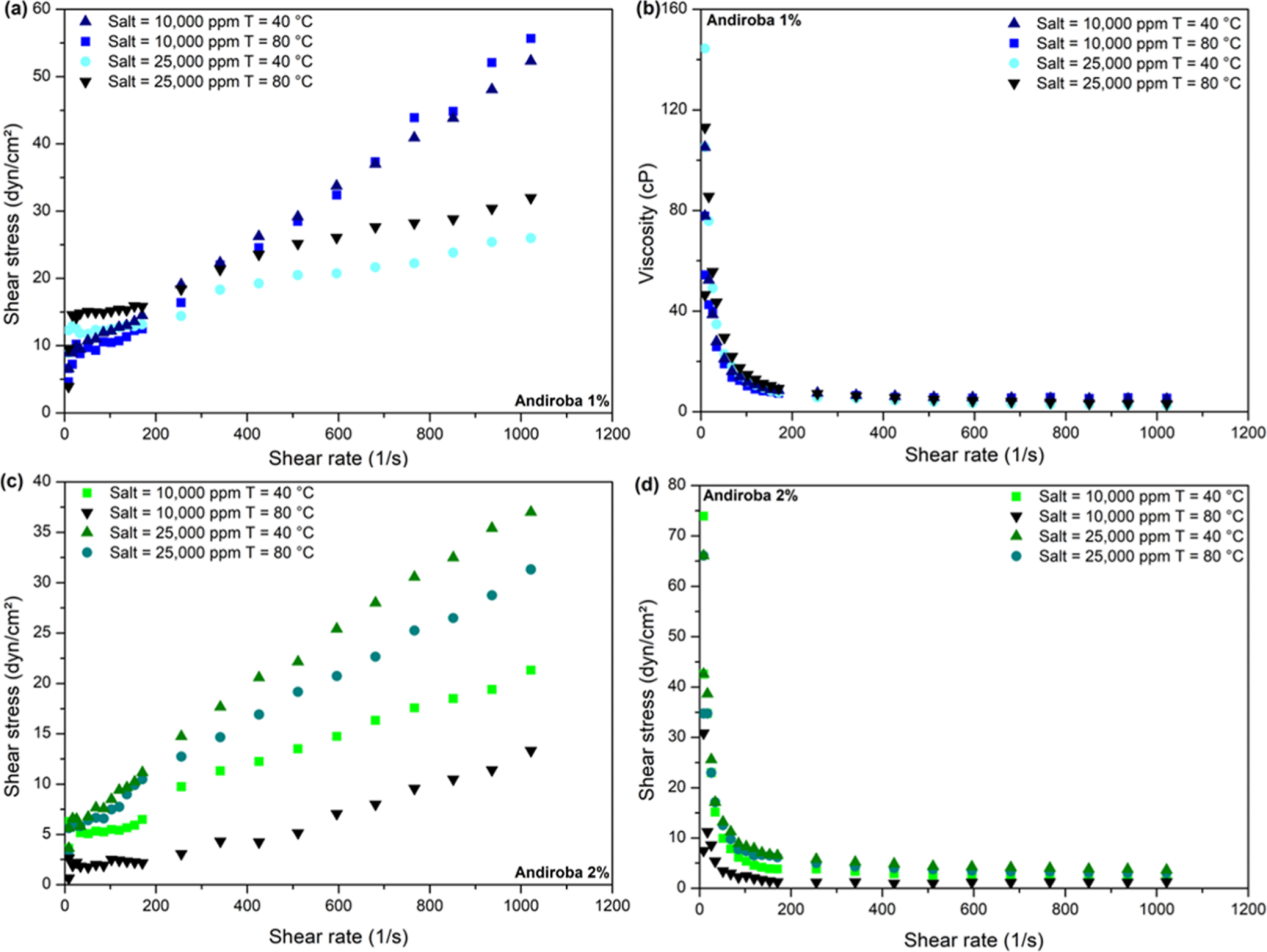
Rheology profiles for: andiroba 1 wt %—shear stress (a) and viscosity (b) curves and andiroba 2 wt %—shear stress (c) and viscosity (d) curves.

The statistical results for modeling of the curves indicate that all models evaluated presented high values of R^2^ and R^2^_adjust_ (>0.90); thus, other statistical analyses were performed to assess the best model that fit the studied curves. The root-mean-square error (RMSE) is used to visualize the discrepancy between the real and the calculated values, therefore, it is a great measure of goodness-of-fit for regression models.^34^ Since lower values of RMSE are desired and considering that the Power Law model showed the greatest values of RMSE in all modeled data sets, it was discharged as a potentially satisfactory model.

The Akaike information criteria (AIC) is another statistical tool that may be employed for model selection, where the best model is the one with the smallest AIC.^35^ By comparing the values of AIC, Tables S4 and S5 indicate that the analyses performed at *T* = 40 °C were better represented by the Bingham Plastic (BP) model (except the one with andiroba = 2 wt % and salt = 25,000 ppm), while at *T* = 80 °C, the Herschel–Bulkley (HB) model had a better adjustment. This shows that the rheological profile of the produced drilling fluids is mainly dependent on temperature, which is consistent with the factorial design analysis since the models for both yield point (YP) and Gel-10 min have temperature as a significant variable.

The implications of the transition from PB to the HB model require a deeper analysis. For both andiroba concentrations evaluated (1% wt and 2% wt), with a salt concentration of 25,000 ppm, and at 80 °C, the flow behavior index of the HB model was found to be n <1, indicating shear-thinning behavior. According to Guo and Liu,^36^ “this shear-thinning property is very desirable in drilling operations because we want low viscosity to reduce the circulating pressure in normal drilling operations, and we want high viscosity during circulation breaks to suspend drill cuttings in the annulus”. Therefore, under highly saline conditions, the HB model describes a fluid with rheological characteristics that are advantageous for routine drilling operations, as it can simultaneously facilitate fluid circulation and promote cutting suspension when flow is interrupted.

In contrast, for low salinity conditions (10,000 ppm) at high temperatures (80 °C), the estimated values of *n* >1 indicate a transition that tended toward shear-thickening behavior. Such behavior is generally undesirable in drilling fluids because viscosity increases with an increasing shear rate, which can result in higher pumping requirements, increased pressure losses, and less efficient hydraulic performance.^37^ These results suggest that, at elevated temperatures, increasing the salt concentration may help preserve a shear-thinning rheological profile, thereby maintaining more favorable flow characteristics for drilling applications.

This interpretation is consistent with the optimum operating conditions predicted by the desirability function (1 wt % andiroba, 14,800 ppm salt concentration, and 78.4 °C), as highlighted in section 3.2.6, which corresponds to a region where the fluid exhibits a balance between adequate rheological performance and operational efficiency.

Figure 6 shows a comparison between the observed values and those calculated by the Bingham plastic model and by the factorial design model. It can be noted that, although the factorial design model did not include temperature as a significant factor for the PV model, the results obtained utilizing the model displayed previously in eq 5 are nearer to the real values. This shows the advantage of the factorial design approach since its estimation is based on process parameters (andiroba and salt concentrations) that were not included in the traditional modeling (Bingham plastic model).

**Figure 6:**
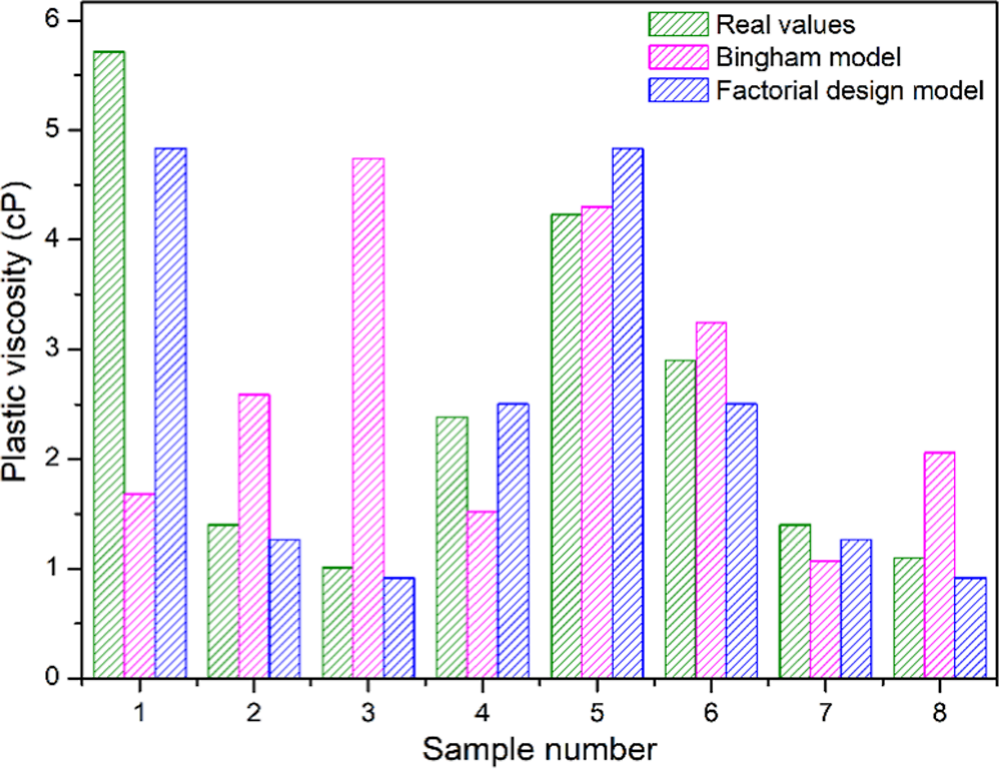
Comparison of plastic viscosity values: real × Bingham model × factorial design model.

Finally, it is well known that, for drilling fluid operations, a fluid that follows the Bingham plastic model is desired because it may indicate possible sources of contamination by evaluating the variation of its parameters, i.e., high values of PV are related to solid contamination, while high values of YP indicate chemical contamination.^38^ Thereafter, one may be more prone to operate under conditions where the drilling fluid follows a behavior more like the Bingham Plastic model. Such conditions can be further improved in future work, where the andiroba additive can still be considered as a possibility for modifying the rheology of drilling fluids, with recommendations for including analysis regarding API fluid loss, filter cake characteristics, thermal stability beyond 80 °C, and comparison of new drilling fluid formulations including commercial additives, such as Carboxymethyl Cellulose (CMC), Polyanionic Cellulose (PAC), and xanthan gum.

### 3.4 Evaluation of Possible Bentonite–Andiroba Mechanism

Bentonite has a lattice structure where an aluminum octahedral sheet is located in between two silica tetrahedral sheets, as a result, this type of clay has a permanent negative charge on the basal plane, while its edge surfaces are pH-dependent.^39^ The point of zero charge (pH_PZC_) for bentonite has been reported in a large range from 2.50 to 8.00.^39,40^ Values of working pH below the pH_PZC_ of a material result in a positively charged surface, while pH > pH_PZC_ implies a negatively charged surface. On the basis of this and considering the results presented in Table 4 (7.65 < pH < 8.29), the edge surfaces of bentonite are expected to be negatively charged on the working pH for this study.

Andiroba seed oil is mainly constituted of fatty acids, such as oleic, palmitic, stearic, and linoleic acids, in the approximate proportion of 52%, 25%, 9%, and 8% of crude oil, respectively, beyond an unsaponifiable portion that includes triterpenes, flavonoids, coumarins, steroids, and limonoids.^41^ Leal et al.^6^ also reported that oleic acid (OA) is the main constituent among the fatty acids of andiroba oil, reaching up to 85% of this oil’s lipidic profile. As for OA, the p*K*_a_ value is 9.85,^42^ where pH < p*K*_a_ presents the molecular form of OA, while pH > p*K*_a_ has higher fractions of its deprotonated form, the oleate anion. Therefore, there are three possible combinations between OA and bentonite: (i) at very low pH (pH < pH_PZC_ and pH < p*K*_a_), the bentonite surface is positively charged, while OA is in its molecular state; (ii) at pH_PZC_ < pH < p*K*_a_, the bentonite surface is negatively charged, while OA is in its molecular state; (iii) at pH > pH_PZC_ and pH > p*K*_a_, the bentonite surface is negatively charged, while OA is in its anionic state (oleate ion).

It is clear that the (iii) option highly disfavors the adsorption of OA into bentonite due to electrostatic repulsion of their negative charges. However, considering the pH values displayed in Table 4 (7.65–8.29), the probable configuration in solution is the (ii) option. The adsorption system of (Molecular-OA + negative-Bentonite) provides a lower electrostatic repulsion than the alternative at higher pH values, favoring the adsorption of OA into the bentonite surface. This is supported by the findings of Resende et al.,^43^ which found that the adsorption of OA into natural bentonites were promoted at pH = 8.00.

Other interactions need to be evaluated, such as hydrophobic interactions, hydrogen bonding, *n*–π, and π–π interactions. Hydrogen bonding (H-bonding) occurs when the adsorbent has an hydroxyl group, while the adsorbate has amine, carboxylic, hydroxyl, and/or phenolic groups (donor–acceptor H-bonding relation) or an aromatic ring (Yoshida H-bonding);^28^ H-bonding may occur in this bentonite–OA system to a limited extent through hydroxyl groups located at edge sites of the adsorbent and adsorbed water molecules, however, since many structural hydroxyl groups are embedded within the bentonite crystal lattice, it is not expected to be the dominant adsorption mechanism.

The π–π and *n*–π interactions generally require the presence of aromatic or other π-conjugated systems. The first one (π–π) usually occurs between aromatic rings of both the adsorbent and the adsorbate, while the second (*n*–π) happens via a donor–acceptor complex between π-electron donors and electron-deficient sites.^44^ Although OA contains a carbon–carbon double bond, neither OA nor bentonite contains aromatic rings or extended π-electron systems capable of promoting significant π–π or *n*–π interactions; thus, these interactions are not expected to play a significant role in the process.

Hydrophobic interactions usually occur when nonpolar groups of the molecules tend to aggregate to reduce their contact with the polar solvent (water). According to Rogers,^45^ the *n*-octanol/water partition coefficient (*K*_ow_) can be used as an indicative of tendency for adsorption of hydrophobic contaminants, in such a way that log­(*K*_ow_) <2.5 indicates low adsorption, 2.5 < log­(*K*_ow_) < 4.0 refers to a medium adsorption, and log­(*K*_ow_) >4.0 expects higher adsorption. Since OA exhibits log­(*K*_ow_) = 7.73, hydrophobic interactions are likely to contribute to the adsorption process. Thereafter, electrostatic and hydrophobic interactions are expected to be mainly involved in the adsorption of OA into the bentonite surface. Figure 7 summarizes the proposed bentonite–andiroba mechanism.

**Figure 7:**
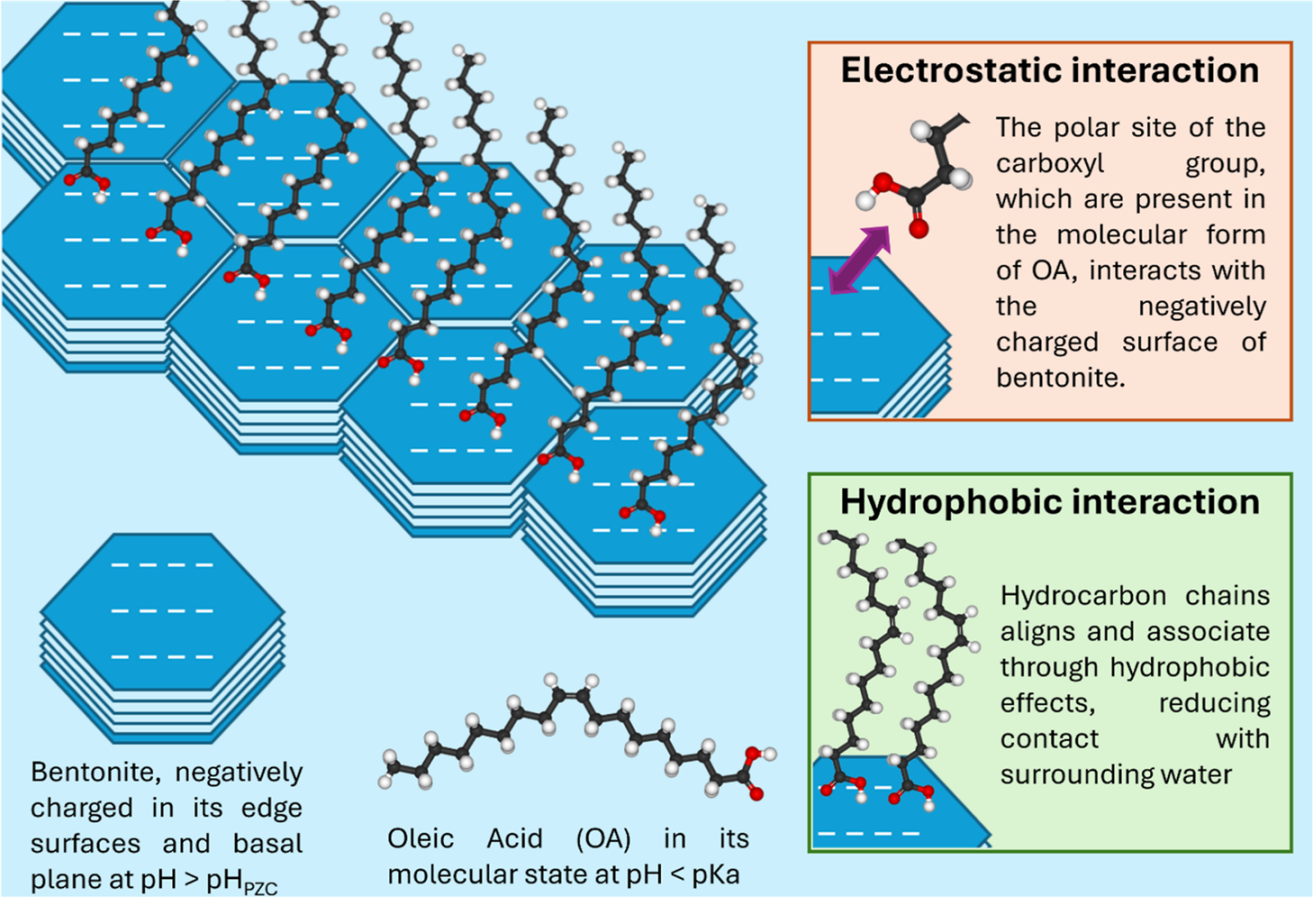
Proposed oleic acid (from andiroba) and bentonite mechanism of interaction.

On the basis of the proposed mechanism, the adsorption of OA onto bentonite particles may modify particle–particle interactions by increasing the hydrophobic character of the clay surface and altering the hydration layer around the particles. Such changes can affect the degree of particle association and the microstructure of the suspension, which may explain the observed variations in rheological parameters. Although the FTIR spectra did not reveal significant changes after the addition of andiroba oil, this may be attributed to the relatively low concentration employed (1 wt %), which limits the detectability of adsorption-induced structural changes. Therefore, adsorption occurring at low surface coverage may substantially affect interparticle interactions and rheological behavior without producing detectable spectral changes. Nevertheless, similar additive concentrations have recently been reported by Ma et al.^8^ in the modification of the lubricating properties of water-based drilling fluids using an oleic acid diethanolamide/graphite composite. While the additives are chemically distinct, their results support the plausibility of oleic-acid-derived compounds interacting with drilling fluid components and influencing macroscopic fluid properties.

## 4 Conclusion

The effect of Amazon biomass as a drilling fluid additive was investigated using physicochemical and rheological parameters. FTIR analysis showed that the drilling fluids had a uniform spectrum due to the high dilution and low concentration of the additive. The andiroba drilling fluid showed rheological parameters consistent with Brazilian legislation, such as PV, YP, pH, and density, revealing a potential use of this biomass.

The quality of a drilling fluid with the andiroba additive can be impacted by other variables present in drilling such as the concentration of salts (NaCl) and the temperature of the well. A 3-factor design was therefore carried out to understand the impact of these variables on parameters such as VP, YP, and gel 10 min. The analysis showed that the ANOVA results and the reduced mathematical models were able to predict the interactions among the parameters that were used in the statistical optimization, identifying the effects that impact the response variables.

Furthermore, according to the results obtained from the response surfaces, a trend was identified in the optimum values for each criterion used (andiroba concentration, NaCl concentration, and temperature) in order to obtain VP, YP, and Gel 10 min values within the legislation. In the response surface graphs, it was clear the great influence and interaction between andiroba and NaCl variables, and regions of maxima could be identified as potentials for drilling operation. The analysis of the results showed that, in order to reach an optimum point of desirability, the optimum values of the input variables need to be 1 wt %, 14,800 ppm, and 78.4 °C, respectively, for andiroba concentration, NaCl concentration, and Temperature, values from which a fluid within the legislation is obtained for the parameters studied (VP, YP, and Gel 10 min).

Rheological analysis showed that the drilling fluid mainly followed a Bingham plastic model at smaller temperatures, while the Herschel–Bulkley model was favored at higher temperatures. In addition, the factorial design model could calculate more satisfactory predicted values for plastic viscosity than the Bingham model, which showed the advantage of employing this methodology. Finally, the interaction between andiroba and bentonite probably occurred mainly through electrostatic and hydrophobic interactions, which correlates with the macroscopic changes in fluid rheology. Therefore, the possible use of an Amazon additive in drilling fluids is an operationally and economically viable alternative, since the fluid produced using andiroba can achieve good operational aspects; besides, it can encourage the Amazonian bioeconomy by using a regional additive.

## Supplementary Material



## Notes

Fisher’s test for complete and reduced models, rheological modeling parameters and statistics for andiroba 1 wt % and 2 wt %, and image of the biomass and Pareto charts (PDF)
