# Supplementary material for: Amazon Bioproduct as a Potential Additive for Water-Based Drilling Fluids: Rheological Analysis and Factorial Design Evaluation
Source: ACS Omega. 2026 Jul 14;11(29):43991–4004. doi: 10.1021/acsomega.6c03697 (PMC13425472; doi:10.1021/acsomega.6c03697)

Amazon bioproduct as a potential additive for water-based drilling fluids: rheological analysis and factorial design evaluation

Thaise do Socorro de Oliveira Araujo[1]; Brenda Fernanda Honorato de Oliveira[2]; Camilo Andres Guerrero-Martin[1]; Emanuele Dutra Valente Duarte[1,3,*]

[1]*Faculty of Engineering of Exploration and Production of Petroleum, Universidade Federal do Pará – Campus Salinópolis, R. Raimundo Santana da Cruz S/N, Salinópolis, Pará, Brazil. Postal code: 68721-000*

[2]*Faculty of Physics, Universidade Federal do Pará – Campus Salinópolis, R. Raimundo Santana da Cruz S/N, Salinópolis, Pará, Brazil Postal code: 68721-000*

[3]*Faculty of Chemistry, Universidade Federal do Pará, R. Augusto Corrêa 01, Belém, Pará, Brazil. Postal code: 66075-110 (current address)*

SUPPORTING INFORMATION

Table S1. Fisher's test for the complete models based on the ANOVA of Table 6. $F_{tabulated}$ is for ($\nu_1 = 6$, $\nu_2 = 3$, $\alpha = 0.05$).

**Source of variation**	**DF[a]**	**PV**				**YP**				**Gel-10min**			
		**SS[b]**	**MS[c]**	$\mathbf{F_{model/error}}$	$\mathbf{F_{tabulated}}$	**SS[b]**	**MS[c]**	$\mathbf{F_{model/error}}$	$\mathbf{F_{tabulated}}$	**SS[b]**	**MS[c]**	$\mathbf{F_{model/error}}$	$\mathbf{F_{tabulated}}$
Regression	6	19.9522	3.3254	10.37	8.94	18.4891	3.0815	22.31	8.94	63.7520	10.6253	27.70	8.94
Residuals	3	0.9621	0.3207			0.4143	0.1381			1.1508	0.3836		
Total	9	20.9144				18.9034				64.9028			

DF[a]: degrees of freedom; SS[b]: sum of squares; MS[c]: mean of squares.

Table S2. Fisher's test for the reduced models.

	PV					YP					Gel-10min				
**Source of variation**	**DF[a]**	**SS[b]**	**MS[c]**	**$F_{model/error}$**	**$F_{tabulated}$ (4, 5, α = 0.05)**	**DF[a]**	**SS[b]**	**MS[c]**	**$F_{model/error}$**	**$F_{tabulated}$ (4, 5, α = 0.05)**	**DF[a]**	**SS[b]**	**MS[c]**	**$F_{model/error}$**	**$F_{tabulated}$ (3, 6, α = 0.05)**
Regression	4	19.3639	4.8410	15.61	5.19	4	17.7963	4.4491	20.09	5.19	3	58.3777	19.4592	17.89	4.76
Residuals	5	1.5505	0.3101			5	1.1071	0.2214			6	6.5251	1.0875		
Total	9	20.9144				9	18.9034				9	64.9028			

DF[a]: degrees of freedom; SS[b]: sum of squares; MS[c]: mean of squares.

Table S3. Regression coefficients for the reduced models.

	PV					YP					Gel-10min				
**Factor**	**RC[a]**	**SE[b]**	**p[c]**	**CL[d] −95%**	**CL[d] +95%**	**RC[a]**	**SE[b]**	**p[c]**	**CL[d] −95%**	**CL[d] +95%**	**RC[a]**	**SE[b]**	**p[c]**	**CL[d] −95%**	**CL[d] +95%**
Mean/Interc.	2.3794	0.1761	0.00004	1.9267	2.8321	1.7240	0.1489	0.00008	1.3413	2.1067	8.0800	0.3298	0.00000	7.2731	8.8869
Andiroba (1)	−0.4960	0.1969	0.0532	−1.0021	0.0101	−0.5325	0.1664	0.0240	−0.9604	−0.1046	−1.8750	0.3687	0.0023	−2.7772	−0.9728
NaCl (2)	−0.6685	0.1969	0.0193	−1.1746	−0.1624	1.1100	0.1664	0.0011	0.6821	1.5379					
Temperature (3)	-	-	-	-	-	-	-	-	-	-					
1 by 2	1.2883	0.1969	0.0012	0.7822	1.7943	−0.6700	0.1664	0.0101	−1.0979	−0.2421	1.3750	0.3687	0.0097	0.4728	2.2772
1 by 3	−0.2608	0.1969	0.2427	−0.7669	0.2453	-	-	-	-	-	−1.3750	0.3687	0.0097	−2.2772	−0.4728
2 by 3	-	-	-	-	-	0.5150	0.1664	0.0270	0.0871	0.9429					

RC[a]: regression coefficient; SE[b]: standard error; p[c]: probability of significance; CL[d]: confidence limit.

The fifth significant number was shown whenever necessary.

Table S4. Statistical results for non-linear modeling of rheology curves found in this study, for andiroba concentration = 1 wt%.

**Model**	**Parameter**	**Statistics**	**Unit**	**Salt = 10,000 ppm T = 40 °C**	**Salt = 10,000 ppm T = 80 °C**	**Salt = 25,000 ppm T = 40 °C**	**Salt = 25,000 ppm T = 80 °C**
**Power law**	K		s^n.dyn/cm²	0.7933	0.3595	4.9525	4.5825
	n		-	0.5929	0.7180	0.2258	0.2720
		R^2	-	0.9627	0.9638	0.8647	0.9226
		R^2_{adjust}	-	0.9590	0.9602	0.8511	0.9149
		RMSE	-	2.7642	3.0163	1.8915	2.0182
		AIC	-	54.0337	58.0489	36.5828	39.5655
**Herschel-Bulkley**	τ_y		dyn/cm²	8.0819	7.1177	10.8206	8.0499
	K		s^n.dyn/cm²	0.0348	0.0155	0.0380	0.7204
	n		-	1.0305	1.1624	0.8680	0.5024
		R^2	-	0.9985	0.9953	0.9683	0.9360
		R^2_{adjust}	-	0.9982	0.9946	0.9632	0.9259
		RMSE	-	0.5641	1.0848	0.9173	1.8385
		AIC	-	−16.1128	13.9684	6.2510	38.2349
**Bingham plastic**	τ_y		dyn/cm²	7.8215	5.7850	11.3043	12.5952
	μ_p		P	0.0430	0.0474	0.0152	0.0206
		R^2	-	0.9984	0.9926	0.9657	0.9016
		R^2_{adjust}	-	0.9982	0.9919	0.9623	0.8918
		RMSE	-	0.5832	1.3598	0.9522	2.2759
		AIC	-	−17.5430	21.4023	5.0101	45.0933

Table S5. Statistical results for non-linear modeling of rheology curves found in this study, for andiroba concentration = 2 wt%.

**Model**	**Parameter**	**Statistics**	**Unit**	**Salt = 10,000 ppm T = 40 °C**	**Salt = 10,000 ppm T = 80 °C**	**Salt = 25,000 ppm T = 40 °C**	**Salt = 25,000 ppm T = 80 °C**
**Power law**	k		s^n.dyn/cm²	0.6805	0.0317	0.5574	0.6257
	n		-	0.4861	0.8571	0.6013	0.5549
		R^2	-	0.9194	0.9260	0.9816	0.9754
		R^2_{adjust}	-	0.9113	0.9186	0.9798	0.9730
		RMSE	-	1.6016	0.9907	1.4663	1.3579
		AIC	-	28.9295	6.8322	24.8697	21.3367
**Herschel-Bulkley**	τ_0		dyn/cm²	4.4480	1.8014	4.3685	4.2484
	k		s^n.dyn/cm²	0.0177	2.3250×10^{-4}	0.0827	0.0698
	n		-	0.9921	1.5597	0.8654	0.8576
		R^2	-	0.9791	0.9877	0.9973	0.9955
		R^2_{adjust}	-	0.9758	0.9857	0.9969	0.9948
		RMSE	-	0.8173	0.4050	0.5643	0.5827
		AIC	-	0.9432	−31.3567	−16.0955	−14.6202
**Bingham plastic**	τ_y		dyn/cm²	4.4756	1.0593	5.4254	5.1516
	μ_p		P	0.0168	0.0107	0.0324	0.0259
		R^2	-	0.9790	0.9624	0.9948	0.9928
		R^2_{adjust}	-	0.9769	0.9586	0.9943	0.9921
		RMSE	-	0.8175	0.7067	0.7775	0.7333
		AIC	-	−2.0072	−8.7057	−4.3129	−7.0033

Figure S1. Andiroba oil, which is obtained from *Carapa guianensis* seeds.

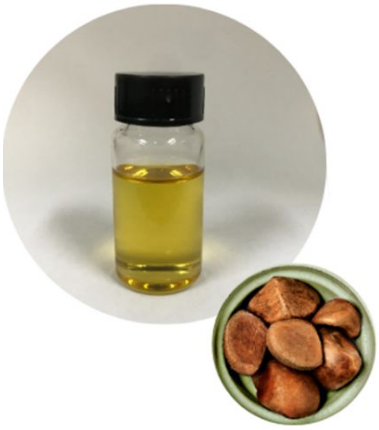

Figure S2. Pareto diagram of the standardized effects for: (a) PV, (b) YP, and (c) Gel-10min.

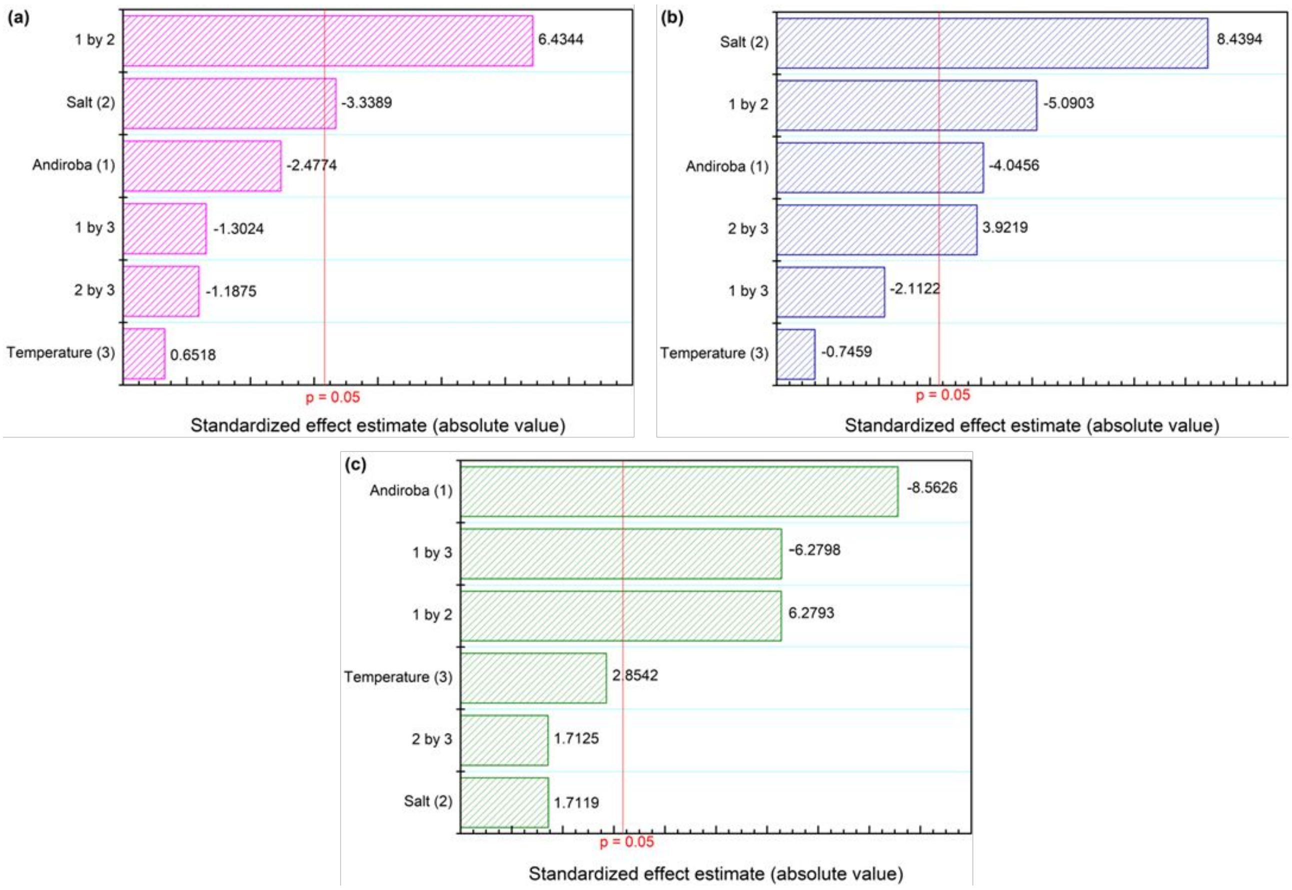

Figure S3. Residual plots for the reduced model of PV: (a) Residuals *versus* observation number; (b) Residuals *versus* predicted values; and (c) Q-Q plot.

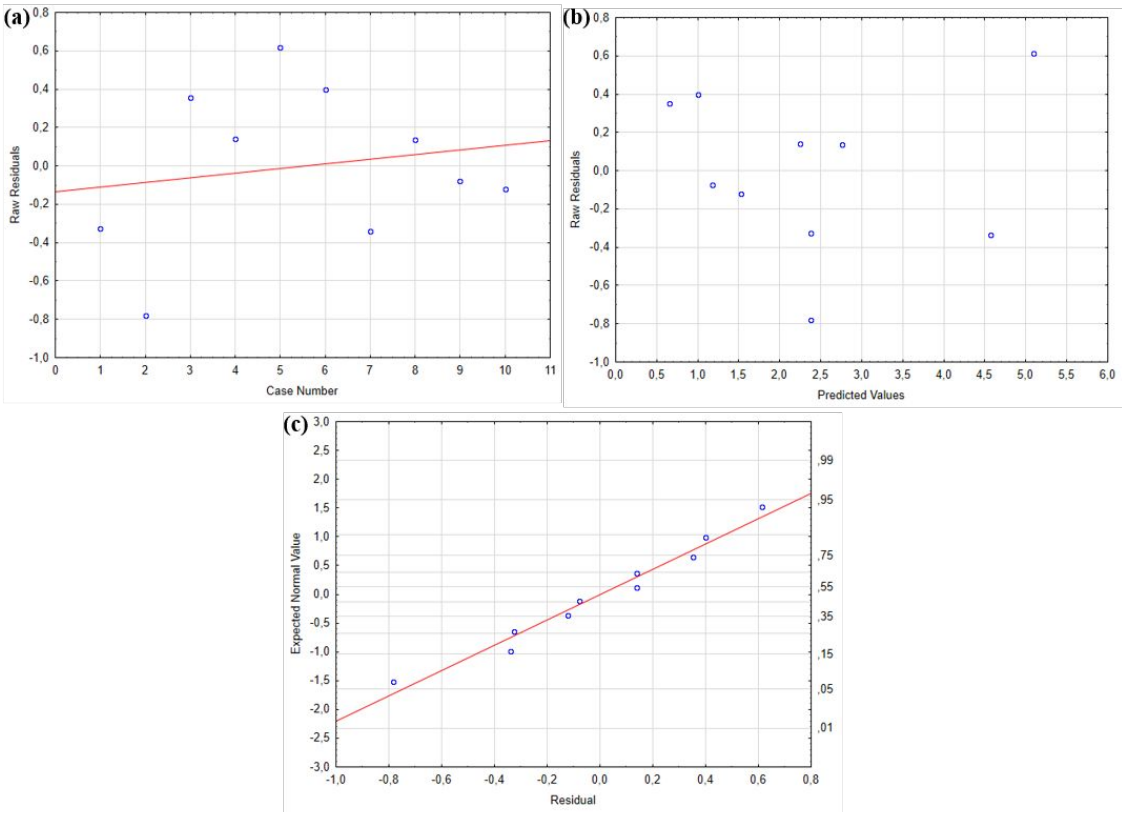

Figure S4. Residual plots for the reduced model of YP: (a) Residuals *versus* observation number; (b) Residuals *versus* predicted values; and (c) Q-Q plot.

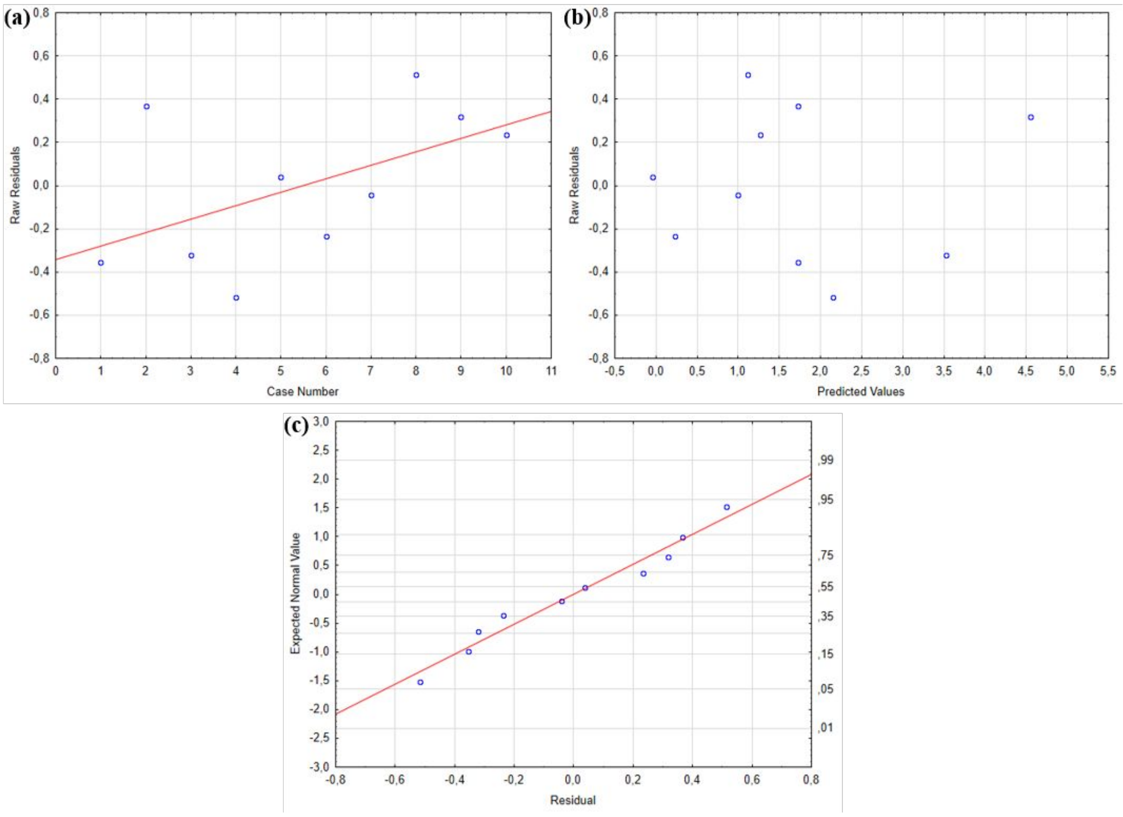

Figure S5. Residual plots for the reduced model of Gel-10min: (a) Residuals *versus* observation number; (b) Residuals *versus* predicted values; and (c) Q-Q plot.

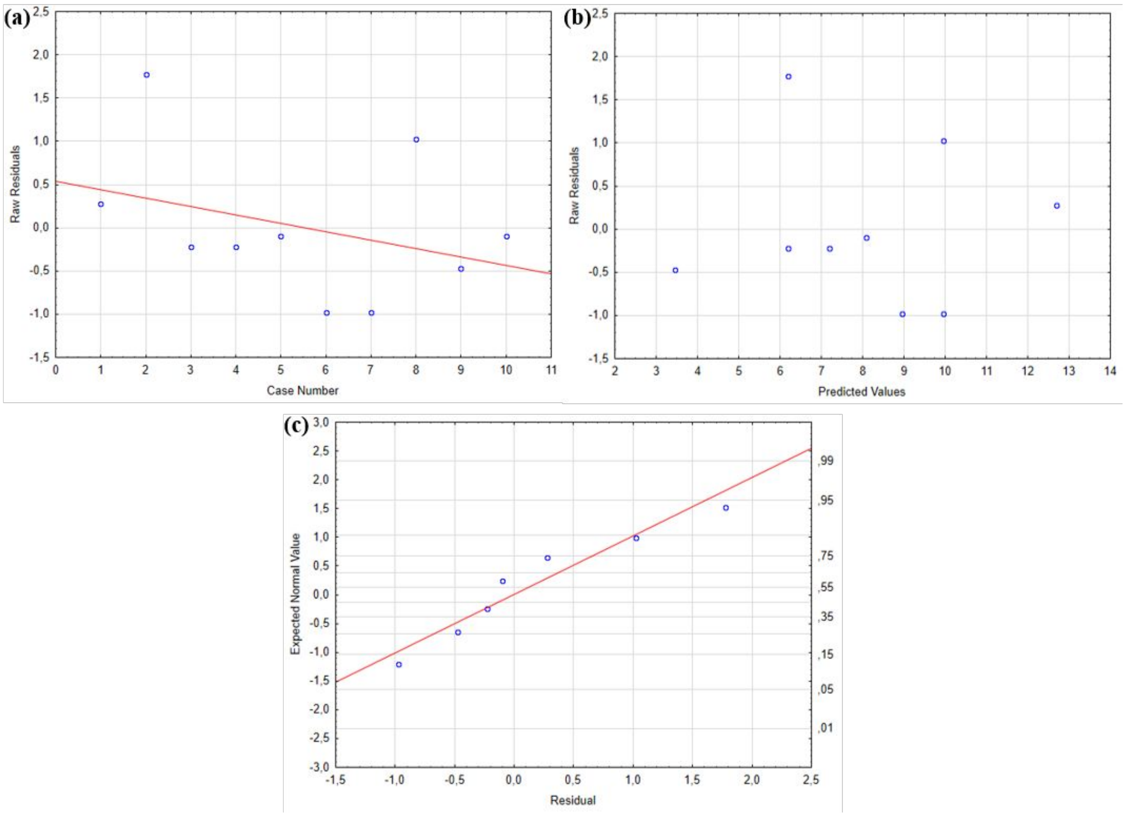

Supplement: Supplementary file 1 [file ao6c03697_si_001.pdf]
